# Improved Flotation Separation of Scheelite from Calcite by Sulfomethylated Kraft Lignin

**DOI:** 10.3390/ma16134690

**Published:** 2023-06-29

**Authors:** Hang Qian, Jinpan Bao, Chuxiong Shen, Dan Wu, Jianshe Wang, Haiqing Hao, Yongsheng Zhang

**Affiliations:** 1 School of Chemical Engineering, Zhengzhou University, Zhengzhou 450001, China; hangqian1996@163.com (H.Q.); bjinpan@163.com (J.B.); scx18015932750@126.com (C.S.); wudan@zzu.edu.cn (D.W.); wangjs07@zzu.edu.cn (J.W.); 2 National Engineering Research Center for Rare Earth, Grirem Advanced Materials Co., Ltd., Beijing 100088, China; 3 GRINM Group Co., Ltd., Beijing 100088, China

**Keywords:** scheelite, calcite, sulfonmethylation, lignin, flotation separation

## Abstract

Low-grade and high-reserve scheelite, which is associated with calcite, has similar surface properties that cause difficulty in separation. In this study, sulfomethylated kraft lignin (SMKL) was used as a novel eco-friendly inhibitor for the flotation separation of scheelite and calcite. The flotation test results showed that 60 mg/L SMKL had a significant influence on depressing calcite flotation, while it had a slight effect on scheelite flotation. Furthermore, it enhanced the WO_3_ grade of the concentrate in the artificial mixed ore to 62.02% with a recovery rate of 80.37%. The contact angle and zeta potential showed that SMKL could effectively decrease the surface floatability of calcite and caused the negative shift of minerals’ surface potential. XPS and DFT calculations revealed that the sulfonic acid group of SMKL had an electron-donating ability and was easily adsorbed on the positively charged surface of calcite, which hindered the adsorption of sodium oleate on calcite. SMKL could separate calcium-bearing minerals with a high efficiency and selectivity, providing a new method for industrial production.

## 1. Introduction

Tungsten is a rare metal with an indispensable strategic position, which has been widely used in the modern industry, national defense, high technology, etc. [1,2,3]. With the long-term over-exploitation of easily separated tungsten resources, the main development target has gradually turned to scheelite resources, whose reserves are high but at low grades. Calcite possesses a similar surface chemistry and similar properties to scheelite, making their separation through flotation very difficult [4]. Room-temperature flotation has become the main method for separating fine-grain scheelite from calcite, given that it is a simple and low-cost process [5,6,7]. Sodium oleate (NaOL) is a conventional trapping agent with active carboxyl groups that enable one to separate practically all minerals by flotation. However, the separation performance of sodium oleate is poor, so that depressants are needed to improve the flotation results [8]. In general, sodium silicate [9], phosphate [10], and carboxymethylcellulose [11] are the main depressants traditionally used in tungsten flotation. However, their use has the disadvantages of requiring a large dosage, with a low selectivity and serious water pollution. Therefore, there is an urgent need to develop a kind of green and efficient depressant for the separation of scheelite from calcite.

Previous reports have revealed that kraft lignin (KL) has the potential to be a good depressant [12]. This is a promising and renewable material mainly derived from the paper industry [13]. However, the physicochemical properties of lignin extracted during paper production experience changes which are viewed as disadvantages: poor water solubility and dispersion, and high viscosity [14,15]. Therefore, it is necessary to modify KL to improve its biocompatibility, so as to expand the range of lignin applications [16,17,18,19]. Indeed, during the sulfomethylation reaction, the aliphatic hydrogen and phenolic hydroxyl groups of sulfated lignin are simultaneously replaced by sulfonic acid groups, with the charge density greatly enhanced [20]. Ying et al. [21] succeeded in introducing the −SO_3_ group into residual lignin in sugarcane bagasse after alkali treatment using sulfomethylation. This resulted in an increase in its hydrophilicity and electronegativity. Vidal et al. sulfomethylated radiata pine kraft lignin and utilized it as a depressant for molybdenite, achieving the successful flotation separation of chalcopyrite and molybdenite [12]. Additionally, Sun et al. indicated that the sulfonic acid groups of calcium lignosulphonate (CLS) interact strongly with the Ca sites on the surface of calcite, chemically adsorbing as Ca−SO_3_. This significantly impedes the subsequent adsorption of sodium oleate on the surface of calcite [22]. To date, the deployment of SMKL as a depressant for flotation separation between scheelite and calcite has not been reported in the literature.

SMKL is a high-value utilization of lignin generated from paper waste streams, which bears good economic benefits and practical significance for environmental protection. In this study, SMKL prepared from kraft lignin was used during flotation tests as a depressant and sodium oleate as the collector in order to explore the separation of scheelite from calcite. The selective inhibition mechanism of SMKL on single minerals was investigated by the contact angle test, zeta potential determination, XPS analysis, and density functional theory (DFT) calculations.

## 2. Materials and Methods

### 2.1. Materials

**Figure 1:**
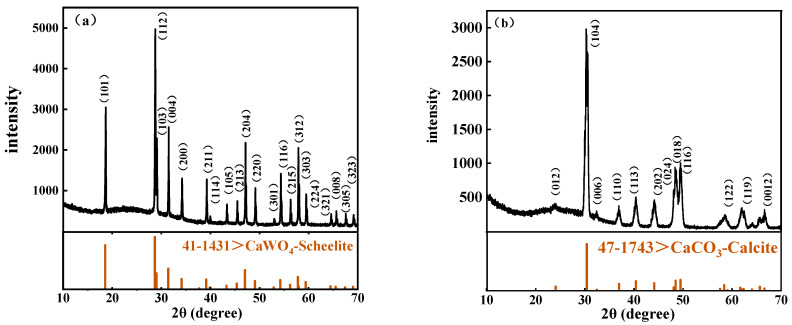
XRD patterns of (**a**) scheelite and (**b**) calcite.

Scheelite and calcite used in this research were sourced from Sichuan and Hunan, China. Before flotation tests, a sample of single minerals was prepared to a suitable particle size. After crushing, the single minerals were crushed to a particle size of less than 0.074 mm using a ceramic ball mill. The analysis by X-ray diffraction (XRD) of a single mineral sample provided the mineralogical composition depicted in Figure 1. The XRD analysis of the mineral samples showed that no impurity peaks were detected in scheelite and calcite, whose high purity met the experiment’s requirements. HCl and NaOH were used to adjust the slurry pH, and kraft lignin (KL) was supplied by Hunan Zhongshan Paper Mill, Hunan, China. Formaldehyde (HCHO), sodium sulfite (Na_2_SO_3_) and sodium oleate (C_18_H_33_O_2_Na) were purchased from Rhawn, Shanghai, China.

### 2.2. Preparation Route of SMKL

**Scheme 1:**
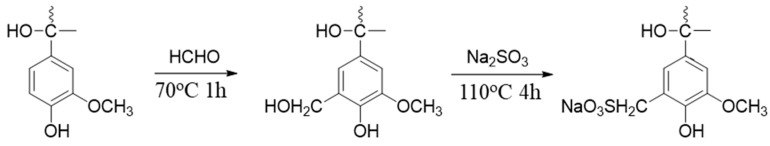
Synthesis equation of SMKL.

A lignin-based depressant (SMKL) was synthesized, as shown in Scheme 1. Firstly, industrial kraft lignin purified by alkaline extraction and acid precipitation was added to 1 mol/L NaOH solution and agitated at ambient temperature for 1 h. Then, the solution was heated to 70 °C, and an appropriate amount of HCHO solution was added to it, with this mixture stirred for 1 h. Subsequently, the reaction temperature was continuously increased until 110 °C, and 70 wt% of Na_2_SO_3_ was added to the mixture. The reaction was allowed to continue for another 4 h. Finally, sulfonyl methylated kraft lignin (SMKL) was obtained after sample purification. The physicochemical properties of SMKL were shown in Supplementary Materials.

### 2.3. Micro-Flotation Tests

**Figure 2:**
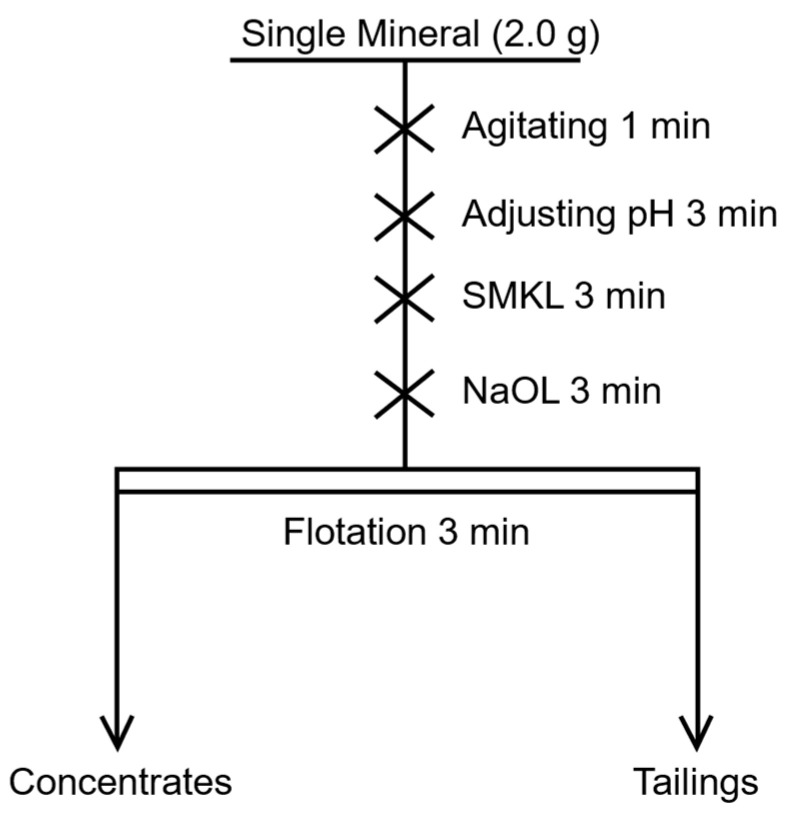
Flow chart of micro-flotation.

The XFG II 5 flotation machine, manufactured by Jilin Exploration Machinery Factory Plant in Jilin, China, was utilized for conducting micro-flotation experiments. The capacity of the flotation machine was 40 mL, with the speed set at 1698 r/min. Specifically, a mixture of 2.0 g sample and 40 mL of deionized water was introduced into a plexiglass pool. The mixture was then stirred for a duration of 1 min to ensure thorough and uniform mixing. To control the pH of the slurry, either HCl or NaOH solution was added accordingly, and depressants and collectors were added sequentially at 3 min intervals. Finally, the foam products formed during the flotation process were collected over a period of 3 min. The resulting concentrate obtained from this collection was dried, weighed, and used to calculate the mineral recovery rate. The average flotation results were determined by repeated micro-flotation tests conducted three times under each group of reagents. The experimental reaction time and the addition sequence of agents are shown in Figure 2.

### 2.4. XRD Tests

XRD analysis was performed using a Bruker D8 Advance X-ray diffractometer. The samples were cut into 2 × 2 cm^2^ sheets, placed flat on a clean carrier table, and scanned using Cu Kα radiation (λ = 1.5418 Å). The test parameters included a tube voltage of 40 kV, tube current of 30 mA, and scanning range of 10–80° with a step size of 10.2°/s. The results were compared with the standard card in the jade v6.0 software.

### 2.5. Contact Angle Tests

The floatability of a mineral is influenced by the contact angle exhibited on its surface. The sample to be tested was produced as a smooth sheet by tablet press. A steady water drop is formed by placing deionized water droplets on a mineral sheet with a micro-injector. A contact angle meter (JY-82C) was used for measuring the contact angle. The contact angles of each sample were measured three times at different locations, and averages were reported in the results. The standard deviation of each test was shown as an error line.

### 2.6. Zeta Potential Measurement

The sample’s zeta potential was determined utilizing a Nano-ZS 90 zeta potentiometer manufactured by Malvern Instruments Ltd., Malvern, UK. To prepare the sample, 20 mg of mineral sample was mixed with 40 mL electrolyte solution (0.1 M KCl) and thoroughly stirred. The suspension was treated by ultrasound and stirred with a magnetic agitator for 10 min, and then NaOH/HCl was used to adjust the pH value and target concentration of the flotation agent. The reaction time and adjustment method were consistent with the method used in the flotation experiment. After the suspension was standing, an appropriate amount of supernatant was taken for several measurements, with the average value taken as the final result.

### 2.7. XPS Tests

A total of 2.0 g of single minerals with a particle size of −2 μm was added to 40 mL of deionized water and stirred well. pH adjuster and SMKL depressant were added sequentially for 15 min. The sample to be tested was then extracted and dried, tested, and analyzed using the Thermo Scientific Nexsa instrument, Waltham, MA, USA.

### 2.8. DFT Calculation

The application of DFT has been extensively used in previous research to elucidate the correlation between surfactant molecules and minerals [23]. In this study, the corresponding structure optimization, charge distribution, and molecular electrostatic potential (MEP) were determined using DMol 3 package implemented in Materials Studio 2019. The exchange-correlation energy in the calculations was approximated using the GGA-PBE functional [24]. For the self-consistent field (SCF) calculations, the convergence criteria were set as follows: 1.0 × 10^−6^ kJ/mol for energy, 1.0 × 10^−5^ Ha for maximum force, 2.0 × 10^−3^ Ha/Å for maximum displacement, and 5 × 10^−3^ Å for displacement.

## 3. Results and Discussion

### 3.1. Micro-Flotation Results

**Figure 3:**
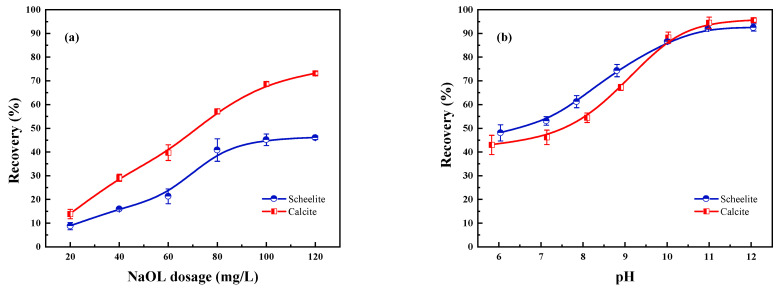
Recovery of single-mineral flotation (**a**) with different dosages of NaOL at natural pH and (**b**) with different pH at c(NaOL) = 90 mg/L.

The effect of NaOL dosage and pH on the recovery of single minerals through flotation is depicted in Figure 3. As shown in Figure 3a, it was evident that increasing the NaOL dosage significantly enhanced the recovery of scheelite and calcite. When the NaOL dosage equaled 80 mg/L, the recovery of scheelite and calcite was 40.81% and 50.14%, respectively. The recovery of scheelite tended to level off when the dosage continued to increase. Figure 3b shows that both the recovery of scheelite and calcite showed an increasing trend as the pH increased from 6 to 12. Their floatability was good in the pH range of 9–12. The above results indicate that achieving an effective separation between scheelite and calcite could not only adjust the NaOL dosage and the pH of the slurry.

**Figure 4:**
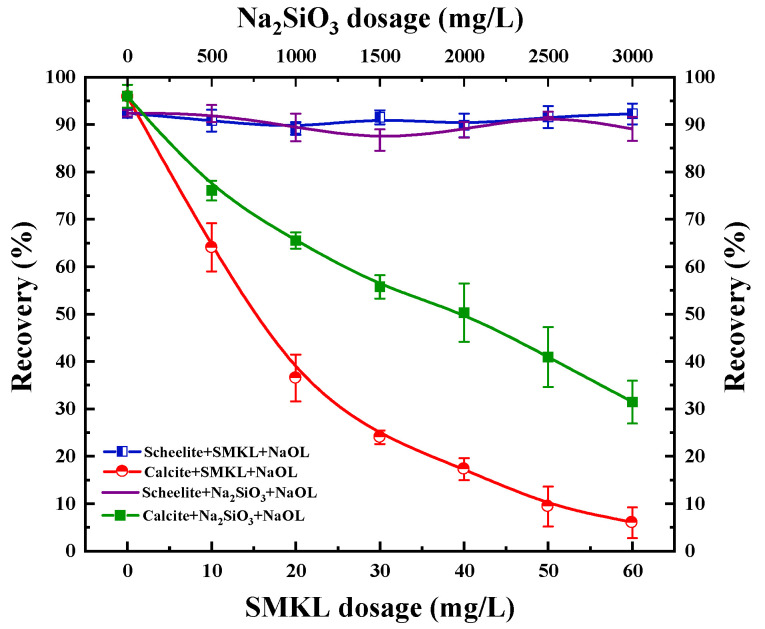
Effect of SMKL and sodium silicate depressant dosage on floatability of single minerals.

The floatability of scheelite and calcite was investigated at pH = 10.5 and a NaOL dosage of 90 mg/L, with different depressant dosages (see Figure 4). After addition of SMKL or Na_2_SiO_3_ as the depressant, the recovery of scheelite was greater than 80% at different dosages. At a concentration of 60 mg/L, SMKL exhibited recovery rates of 92.34% for scheelite and 5.98% for calcite. Meanwhile, at a Na_2_SiO_3_ dosage of 3000 mg/L, the recovery of scheelite was 89.11%, and that of calcite was only reduced to 31.44%. With the increase of the depressant dosage, calcite recovery decreased gradually, but the decrease of calcite recovery by SMKL was much larger than that of Na_2_SiO_3_, while the dosage of SMKL was much lower than that of Na_2_SiO_3_. This indicates that SMKL is a promising depressant.

In addition, flotation experiments were conducted on a binary mixed ore (m:m = 1:1), and it was preliminarily confirmed that scheelite could potentially be separated from calcite using SMKL. From Table 1, it can be seen that without SMKL, the WO_3_ grade of the concentrate is 39.77%, which is almost the same as the original mixed feed. However, with the addition of 40 mg/L of SMKL, the WO_3_ grade of the concentrate increased to 62.02%, with a recovery rate of 80.37%. This indicates that the use of SMKL as an inhibitor can achieve flotation separation between scheelite and calcite.

### 3.2. Contact Angle Analysis

**Figure 5:**
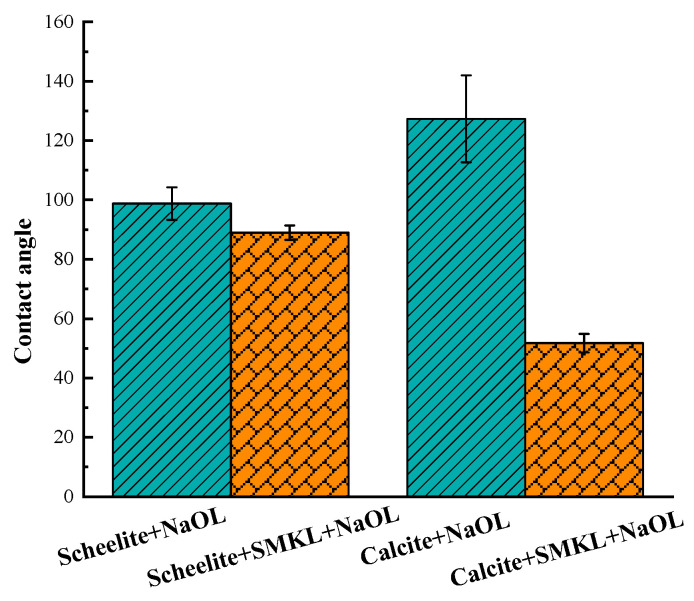
Contact angle of single minerals treated with different chemicals.

Figure 5 displays the contact angles of minerals when exposed to various reagents under a pH of 10.5. After being treated with NaOL, the contact angles of scheelite and calcite were 98.8° and 127.3°, respectively. This showed that their hydrophobicity and floatability significantly increased. The contact angle was 89.0° after treatment of scheelite and calcite with SMKL and NaOL, revealing the fact that SMKL acted weakly upon the post-adsorption of NaOL on the surface of scheelite. In contrast, the contact angle of calcite treated with SMKL and NaOL was reduced to 51.7°, revealing the fact that its hydrophilicity increased. The above results show that the adsorption of SMKL on the surface of calcite was greater than for scheelite, so that it eased their separation through flotation. Consequently, the flotation of calcite was selectively inhibited, as shown by the results from the flotation tests.

### 3.3. Zeta Potential Analysis

**Figure 6:**
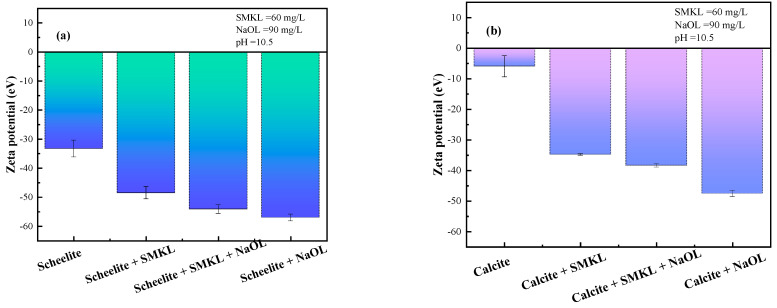
Zeta potential of (**a**) scheelite and (**b**) calcite.

Zeta potential is an important tool used for elucidating the mechanism of adsorption of reagents on minerals’ surfaces [25,26]. As shown in Figure 6, the adsorption behavior of SMKL and NaOL on the surfaces of two minerals at pH 10.5 was investigated via the zeta potential’s measurement. After treatment of the minerals under consideration with SMKL, negative shifts of zeta potentials were observed, indicating that SMKL was adsorbed on both scheelite and calcite in the form of anions. The calcite zeta potential showed a negative shift of 28.9 mV (Figure 6b), greater than the 15.2 mV measured in the case of scheelite (Figure 6a). This revealed that SMKL adsorption on calcite had a higher intensity compared to scheelite. The addition of NaOL decreased the calcite zeta potential to only 3.6 mV. This value was lower compared to the value of 5.6 mV observed in the case of the scheelite zeta potential. The above result shows that NaOL adsorption on calcite was weaker than on scheelite. Therefore, an increased SMKL adsorption and decreased NaOL adsorption on calcite could selectively help to inhibit its flotation.

As can be seen from Figure 6a, it was observed that the zeta potentials of scheelite at pH 10.5 were similar to those observed when scheelite was treated with either NaOL or with SMKL + NaOL. This reveals that SMKL has little effect on the adsorption of NaOL on the scheelite’s surface. Conversely, the zeta potential measured after calcite treatment with NaOL increased by −47.6 mV. This value is more negative than the one measured when calcite was treated with both SMKL and NaOL. The above result implies that the early strong adsorption of SMKL on calcite could inhibit the adsorption of NaOL. This also means that the treatment of calcite with SMKL could result in a weak adsorption of NaOL. These results were consistent with the flotation results presented in Figure 4.

### 3.4. XPS Analysis

XPS analysis is enabled through bonds energy measurements that analyze the chemical environment of multiple elements on a mineral’s surface. In this article, XPS tests were performed to confirm the interaction mechanism between SMKL and the minerals [27,28]. Based on the atom content provided in Table 2, it can be stated that the atomic ratio of Ca 2p, W 4f, O 1s, and C 1s on the surface of scheelite did not change significantly after its treatment with SMKL. The above result reveals that SMKL acted weakly on the surface of scheelite. After treatment with SMKL, the S 2p ratios of scheelite and calcite were increased by 0.06% and 0.26%, respectively, revealing the selective adsorption of SMKL on calcite. The ratio of C 1s in calcite increased after being treated with SMKL, while the ratio of O 1s and Ca 2p decreased. This may be induced by the selective adsorption of organic complexes formed by SMKL and Ca^2+^ on the mineral surface.

**Figure 7:**
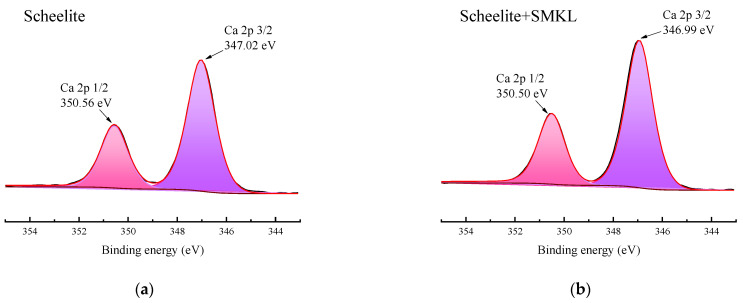

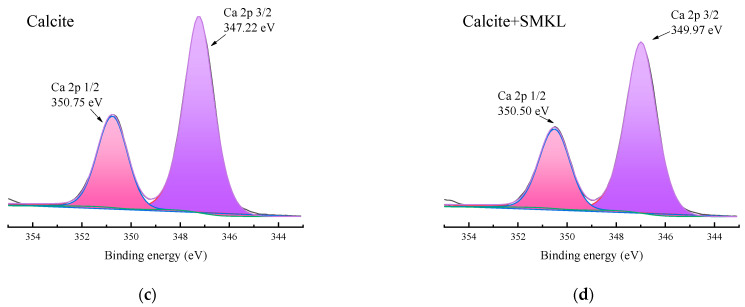
XPS spectra of Ca 2p on (**a**,**b**) scheelite and (**c**,**d**) calcite surfaces (c_SMKL_ = 70 mg/L).

High-resolution spectra of calcium measured on the surface of minerals under study enabled one to highlight the adsorption sites of reagents used [29,30]. The high-resolution XPS of Ca 2p on scheelite and calcite are depicted in Figure 7. As can be seen from Figure 7a, the Ca 2p energy bands of scheelite at 347.02 eV and 350.56 eV correspond to Ca 2p 3/2 and Ca 2p 1/2, respectively [31]. After treatment of scheelite with SMKL, the observed peaks shifted to 346.99 eV and 350.50 eV, respectively. These small changes within the instrument error range indicated the weak adsorption of SMKL on scheelite. The two peaks of calcite Ca 2p were Ca 2p 3/2 at a binding energy of 347.22 eV and Ca 2p 1/2 at a binding energy of 350.75 eV, respectively [31]. The binding energy of calcite treated with SMKL was shifted to 346.97 eV and 350.50 eV on Ca 2p 3/2 and Ca 2p 1/2, respectively, with a binding energy shift of −0.25 eV. This showed that SMKL could adsorb on the surface of calcite, with a greater influence on its floatability. As suggested in previous studies, calcium ions from the calcite surface are adsorption sites for lignosulfonate, which also chemically interacts with Ca^2+^ obtained due to the addition of calcium hydroxide [32,33]. When combining findings from previous reports to those from the obtained XPS results, it can be inferred that −SO_3_ from SMKL chemisorbed with Ca^2+^ from the calcite surface.

### 3.5. DFT Calculation

**Figure 8:**
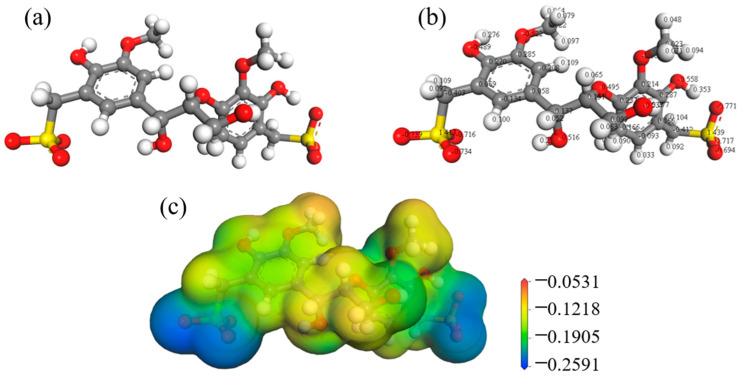
Optimized SMKL (**a**) structure, (**b**) charge distribution, and (**c**) electrostatic potential.

In this article, DFT was applied to elucidate the surfactant molecules’ properties and assess their adsorption capability [34]. Figure 8 depicts the optimized structure, charge distribution, and molecular electrostatic potential (MEP) of SMKL’s anion. From the charge distribution of SMKL, it can be stated that negative charges were mainly concentrated on sulfonic acid groups (−SO_3_), hydroxyl groups, and the oxygen atoms of methoxy groups. The natural charges of the oxygen atoms in the hydroxyl and methoxy groups are approximately −0.489 e and 0.522 e, while the oxygen atom connecting the sulfonic acid group has a lower natural charge, reaching −0.771 e. The three oxygen atoms (−0.739 e, −0.734 e, and −0.716 e) in the sulfonic acid group have lower charges. This reveals that they were most likely to lose electrons. Electron-donating ability is involved in the SMKL anion adsorption’s reaction [35].

In addition, the MEP measurement at a point of the molecule provided valuable information for assessing the reactivity of the molecule to positively or negatively charged sites. The region with the highest concentration of negative charges was highlighted by blue color, as opposed to the red color region, which stood for areas with the greatest positive charges [36]. In the MEP, the blue hue reflected a more negative electrostatic potential, with the major negative electrostatic potential primarily situated around the oxygen from the −SO_3_ group. This suggests that −SO_3_ was chemically active and likely to engage in coordination reactions with metal ions to produce complexes [33,37]. Relying on the DFT calculations, the anionic group (−SO_3_) of the SMKL depressant produced an electrostatic attraction with the calcite surface, which could further improve scheelite separation through flotation in the presence of calcite.

## 4. Conclusions

Within the pH range of 9–12, the flotation tests’ results showed that the presence of SMKL depressant could enable the maintenance of a good floatability of scheelite, while significantly reducing the recovery rate of calcite. Additionally, a flotation experiment was conducted on a mixed ore of scheelite and calcite, resulting in a concentrate with a WO_3_ grade of 62.02% and a recovery rate of 80.37%. The variation in the zeta potential evidenced the interaction of the SMKL depressant with the surface of the studied minerals. SMKL brought about more negative shifts in zeta potential on the calcite surface when compared to the scheelite. After the SMKL depressant’s interaction with both minerals, the binding energy of Ca 2p on the surface of calcite decreased when compared to scheelite, for which the effect was revealed to be minimal. The chemical adsorption of SMKL on the surface of calcite hindered the adsorption of NaOL, and this phenomenon enabled the selective separation of scheelite and calcite through flotation. DFT calculation showed that the electron donor of SMKL was mainly concentrated on −SO_3_ groups, which electrostatically interacted with the calcite surface’s Ca^2+^. This study explored the use of SMKL as a depressant in the separation of scheelite and calcite, illustrating a new approach to separate calcium-bearing minerals.

## Figures and Tables

**Table 1:** The results of binary mixed ore (pH ≈ 10.5; c(NaOL) = 90 mg/L).

Parameters	Products	Yield/%	WO_3_ Grade/%	WO_3_ Recovery/%
SMKL 0 mg/L	Concentrate	89.54	39.77	91.24
	Tailing	10.46	32.67	8.76
	Feed	100	39.03	100
SMKL 40 mg/L	Concentrate	50.58	62.02	80.37
	Tailing	49.42	15.50	19.63
	Feed	100	39.03	100

**Table 2:** The atomic concentrations on mineral surfaces.

Mineral	SMKL	Element (Atomic %)				
		C 1s	O 1s	Ca 2p	W 4f	S 2p
scheelite	before	24.46	52.74	11.45	11.34	
	after	24.73	52.82	11.22	11.17	0.06
calcite	before	33.11	50.47	16.42		
	after	35.62	49.32	14.79		0.26

## Supplementary Materials

The following supporting information can be downloaded at: https://www.mdpi.com/article/10.3390/ma16134690/s1, Figure S1: Infrared spectra of KL and SMKL; Table S1: the physical and chemical properties of KL and SMKL.
