# Supplementary material for: Improved Flotation Separation of Scheelite from Calcite by Sulfomethylated Kraft Lignin
Source: Materials (Basel). 2023 Jun 29;16(13):4690. doi: 10.3390/ma16134690 (PMC10342449; doi:10.3390/ma16134690)
Supplement: Supplementary file 1 [file materials-16-04690-s001.zip › materials-2439175-supplementary.pdf]

materials

MDPI

Supplementary Materials

Improved Flotation Separation of Scheelite from Calcite by Sulfomethylated Kraft Lignin

Molecular weight and degree of sulfonation are important indicators for evaluating the performance of sulfomethylated lignin. Table S1 presents the molecular weights and major functional group contents of KL and SMKL.

Table S1. the physical and chemical properties of KL and SMKL.

	**-SO_3 group（mmol/g）**	**M_w (Da)**	**M_n (Da)**	**PDI (M_w/M_n)**
KL	-	4920	3570	1.38
SMKL	2.34	5216	3852	1.35

Infrared spectroscopy was performed to investigate the functional group changes in the sulfomethylation reaction of the SMKL sample. As shown in Figure S1, two well-resolved peaks are observed at 2933 cm^{-1} and 2845 cm^{-1}, corresponding to the C-H stretching vibrations of the CH_2 and CH_3 groups in lignin. The shape, wavelength, and intensity of these peaks remain unchanged between KL and SMKL. The absorption peak observed at 1703 cm^{-1} in the spectrum of KL is attributed to the C=O stretching vibrations of carboxyl, carbonyl, and acetyl groups, which are reaction sites during the sulfomethylation process. The absorption peak at 1365 cm^{-1} is caused by in-plane deformation vibrations of phenolic hydroxyl groups and CH_3 groups, which result from the cleavage of β-O-4 and α-O-4 bonds in lignin during the sulfation pulping process. The absorption peak at 1270 cm^{-1} corresponds to the C-O stretching and linkage of methoxy groups on the syringyl unit. The specific vibration peaks at 1270 cm^{-1}, 854 cm^{-1}, and 816 cm^{-1} are characteristic of syringyl unit structures, while no specific vibration peaks were observed for the guaiacyl unit (1326 cm^{-1}, 1115 cm^{-1}, and 825 cm^{-1}). This result confirms that the sulfated lignin used in this study is predominantly composed of syringyl unit structures. Comparison between SMKL and KL reveals a significant weakening of these absorption peaks after sulfomethylation. The peak at 1033 cm^{-1} corresponds to the in-plane deformation of aromatic C-H, C-O deformation in primary alcohols, C-H stretching, and S=O stretching, which are still present after chemical modification. KL exhibits absorption peaks attributed to the sulfonic acid group, which may have been introduced into the lignin structure during the sulfation pulping process. Finally, the characteristic peak at 630 cm^{-1} in the SMKL sample corresponds to the C=S bond and is also related to the S-O groups, which increase after sulfomethylation. Additionally, the peak at 495 cm^{-1} after sulfomethylation is associated with the sulfonic acid groups grafted onto the lignin structure [1].

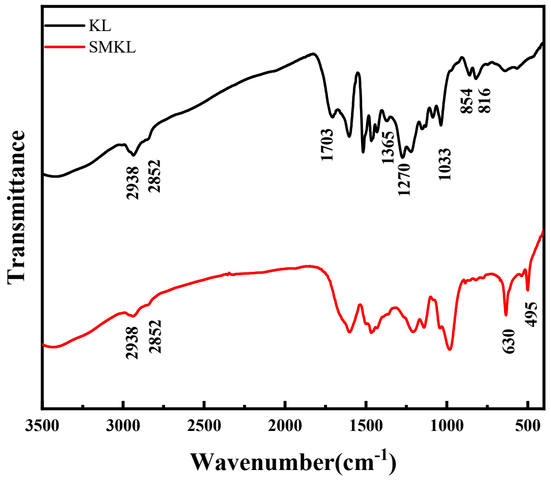

Figure S1. Infrared spectra of KL and SMKL.

Reference

1. Vidal, C.; Carrillo-Varela, I.; Reyes-Contreras, P.; Gutierrez, L.; Mendonça, R. T., Sulfomethylation of radiata pine kraft lignin and its use as a molybdenite depressant in selective chalcopyrite-molybdenite separation by flotation. *BioResources* **2021,** 16, 5646–5666.
